# The Validation of the Revised Illness Perception Questionnaire in Cancer Patients: An Exploratory Factor Analysis for Further Psychometric Evidence

**DOI:** 10.1002/brb3.71603

**Published:** 2026-07-16

**Authors:** Maryam Amini Fasakhoudi, Hossein Karsazi, Asma Shahi, Narin Beyraghdar, Mohammad Noori

**Affiliations:** ^1^ Department of Clinical Psychology, School of Medicine Shahid Beheshti University of Medical Science Tehran Iran; ^2^ Department of Clinical Psychology, School of Medicine Tabriz University of Medical Science Tabriz Iran; ^3^ Master of Health Psychology, School of Behavioral Sciences and Mental Health (Tehran Institute of Psychiatry) Iran University of Medical Science Tehran Iran; ^4^ Department of Psychology Islamic Azad University South Tehran Branch Iran; ^5^ Department of Clinical Psychology, School of Medicine Shahid Beheshti University of Medical Sciences Tehran Iran

**Keywords:** exploratory factor analysis, illness perceptions, reliability, validation

## Abstract

**Objective:**

Applying the Leventhal's model would make it possible to highlight the cognitive and emotional representations of adults with cancer and then to propose specific interventions to improve their adherence. This paper was conducted aiming at the validation of the revised illness perception questionnaire (IPQ‐R) based on an exploratory factor analysis (EFA) in cancer patients.

**Design:**

The IPQ‐R was administered to 300 outpatients referred during a 9 months period. In the present study, we performed an EFA to examine the construct validity of the instrument. In addition, the concurrent validity of the IPQ‐R with the positive and negative affect schedule, depression, anxiety and stress scale‐ 21 items and 36‐item Short‐Form Health Survey questionnaires was evaluated. Reliability was calculated using Cronbach's alpha, McDonald's omega, and test‐retest methods.

**Results:**

EFA of the beliefs and causes subscales revealed seven dimensions and three factors, respectively. With the difference that in the causes subscale, instead of the four factors of the original study, three factors were extracted. The validity and reliability of the identity subscales and beliefs overall were rated as good to excellent, but the concurrent validity of the Causes subscale was in the factor of psychological attributions and risk factor high and low, respectively, and for the substance use factor, it also did not obtain any correlation. For reliability, the risk factors received a low score.

**Conclusion:**

Adaptation of the IPQ‐R in Iranian culture and an adult population suffering from cancer disease made it possible to use it for this particular population.

## 1 Introduction

The diagnosis of cancer is associated with various psychological problems during and after acute treatment, and the individual perceptions and beliefs about the illness seem to be important for illness management (Rourke et al. 2007). Thus, in order to develop effective interventions to address psychological distress among cancer patients, it is important that we understand the types of cognitions that are associated with higher levels of distress among this population (Dempster et al. 2011a). The Common‐Sense Model of Illness Representation (CSM) is an influential theoretical model of self‐regulation that links illness management and coping strategies related to health behavior. This model postulates that individuals create cognitive representations of their illness when being confronted with it, and these illness representations are the important target for screening, planning effective interventions, and improving patient outcomes (Leventhal 1980).

The IPQ revised version's main advantage is that, in addition to assessing the cognitive components of illness representation, it is also able to examine the emotional representation of illness. It should be noted that the IPQ‐R is not only the indicator of patients’ general mood but also provides an assessment of the emotional responses generated by the illness (Pasternak et al. 2021). This finding that cognitive dimensions of illness representation can be separated from both emotional representations and positive and negative emotional characteristics, has particular importance (Moss‐Morris et al. 2002).

Components of disease representation in Leventhal's self‐regulatory model are reflected in three sections of the Revised Illness Perception Questionnaire (IPQ‐R). The first section is the identity subscale with 14 questions (e.g., pain, nausea, breathlessness, fatigue, and upset stomach) and addresses the issue of whether the patient has experienced any of the symptoms during the illness and whether he can accurately identify each of them. The second section is the perceptions of the patient, which includes seven dimensions (timeline–acute/chronic, consequences, personal control, treatment control, illness coherence, timeline–cyclical, and emotional representations). The third section consists of 18 questions about the cause of the disease (e.g., hereditary, stress, and pollution in the environment), and the patient answers them based on his/her beliefs about the factors that may have caused the disease (Pasternak et al. 2021). The identity and causes subscales are psychometrically different from the beliefs scale and comprise items, which vary in number and content by patient sample (Moss‐Morris et al. 2002). As a result, researchers should be able to modify the causal and identity scales in order to correspond with particular illnesses and populations (Pasternak et al. 2021). The findings of a meta‐analysis revealed that IPQ‐R subscale reliability varied based on sample characteristics. Therefore, adapting subscales in various samples could improve the measurement of illness perception constructs (Rivera et al. 2024). Again, this scale should be adapted to the specific disease and is conducted a new exploratory appraisal of the factorial structure (Oudin Doglioni et al. 2022). Also, investigations indicate cultural differences in the perception of the same disease, especially its symptoms and causes. For example, this can be clearly observed in research on cervical cancer representation (Chen et al. 2020).

In this regard, Dempster and McCorry (2012) conducted a confirmatory factor analysis (CFA) on esophageal cancer survivors and confirmed that the IPQ‐R has a seven‐factor structure. Ashley et al. (2013) assessed the psychometric properties of the IPQ‐R using data of patients with breast, colorectal, and prostate cancers. In this study, the authors conducted CFA and Rasch analysis and confirmed the IPQ‐R factor structure. In both analyses, items 12, 18, and 24 exhibited cross‐loading and were removed. A CFA among Danish colorectal cancer patients indicated no good comparative fit with the Illness Perception Questionnaire–Revised, but the results of exploratory factor analysis (EFA) supported a seven‐factor structure (Hvidberg et al. 2014). The results of a study demonstrated that IPQ‐R can be successfully used in the Polish cultural context as a useful instrument for assessing cognitive and emotional representations linked to illness in cancer patients (Pasternak et al. 2021). In another study, the IPQ‐R was reported as a reliable and valid tool for assessing illness perception among Chinese patients suffering from cervical cancer (Chen et al. 2020). The results of a study in Portugal indicated that IPQ‐R is a reliable and valid instrument to evaluate the cognitive and emotional representation of cancer patients. In this study, the substitution of some items was suggested for the illness identity subscale. Further, the correlation among the different dimensions of the beliefs subscale showed that the theoretical bases of IPQ‐R are confirmed in investigation of the cancer illness. Result of the CFA also extracted four factors for the causality subscale, which better explained the variance of the answers (Santos et al. 2003). Factor analysis of the IPQ‐R Greek version revealed that the consequences and emotional representations of the beliefs subscale loaded on one factor. The causal subscale also revealed a different structure from the original questionnaire, which could indicate cultural differences in understanding illness causation. In this study, predictive validity was tested by regressing BDI scores on IPQ subscale scores. Inter‐relationships between IPQ‐R subscales were also examined by computing Pearson's correlation coefficients. The Cronbach's alpha and test‐retest reliability demonstrated satisfactory internal consistency for the IPQ‐R subscales (Giannousi et al. 2010). In a study conducted on 203 Chinese breast cancer survivors, five causal factors were extracted after removing one item, accounting for 68.02% of the variance in total. As well as, an acceptable fit with the data for the seven‐factor model was obtained. Coefficients of Cronbach's α and composite reliability were also acceptable (Huang et al. 2019). The results of the analysis of data obtained from 660 English women referred for cervical cancer screening provided evidence of a confirmatory analysis for seven factors of the IPQ‐R that a theoretically predictable pattern of relationships among the representation dimensions was evident. In particular, constructs of two dimensions of control and illness coherence were negatively related to other illness representation constructs. In addition, factor intercorrelations supported the discriminant validity of the constructs, and the factors demonstrated satisfactory composite reliability (Hagger and Orbell 2005). The results of a validation study defended the modified IPQ‐R as a promising instrument for measuring symptom representations among Danish colorectal cancer patients (Hvidberg et al. 2014). Research has highlighted a strong association between illness perceptions and health‐related outcomes in cancer survivors (Herzog et al. 2024). In line with these findings, in adult cancer survivors, it became revealed that more negative illness perception is associated with higher levels of depression and anxiety (Dempster et al. 2012) and lower levels of health‐related quality of life (Schoormans et al. 2020; de Rooij et al. 2018). Dempster et al. (2011b) conducted a longitudinal study on a sample of adult esophageal cancer survivors and found that changes in illness perceptions over the course of 1 year contributed to changes in anxiety and depressive symptoms. The results of another study on adult patients with recently diagnosed head and neck cancer revealed that illness perceptions are associated with several dimensions of quality of life, such as physical, emotional, and social functioning. Namely, patients with increased attention to symptoms and recurrence were more likely to engage in self‐blame, had a stronger emotional reaction to the illness, and had lower QOL scores (Scharloo et al. 2005). The results of a study revealed that dimensions of illness representations are associated with chronic cancer pain in breast cancer survivors. Further, having a strong illness identity and health‐related quality of life were to be independent, significant predictors of pain severity (Langford et al. 2024).

Illness consequences are directly influenced by illness perception, although the pattern of this association is dependent on the type of disease, and the IPQ‐R developers “have always encouraged researchers to adapt the scale to their particular illness and research setting” (Hagger and Orbell 2003). Likewise, the illness perception among cancer patients is related to their cultural background (Dein 2004). Furthermore, although studies have validated the IPQ‐R for patients with cancer, most were conducted in Western populations. Therefore, the aim of this study is to explore the cultural adaptation and the psychometric properties of the IPQ‐R on Iranian cancer patients.

## 2 Methods

### 2.1 Participants

The study design is psychometric, in which a cross‐sectional method is used to collect data. The original English version of the IPQ‐R was translated into Persian by a fluent speaker of both English and Persian languages. Then, the Persian version was translated back into English by a different translator who had no prior knowledge of the original version. The differences in meaning were identified and reviewed by a third translator. The first step related to translation equivalence that was analyzed was linguistic and translation errors that were conducted for all sections of the IPQ‐R questionnaire. For instance, “sleep difficulties,” “joint stiffness,” and “eye injury” were translated inconsistently, albeit correctly in a linguistic sense. In order to verify the content's accuracy, the IPQ‐R's Iranian translation was then subject to content‐qualitative evaluation (Polit and Beck 2020) by a team of specialists from various fields and with linguistic qualifications (a clinical psychologist, a health psychologist, a cancer intensive care nurse, a neurologists, and an endocrinologist). In such a way, 0 indicated “incomprehensible and questionable,” 1 indicated “hard,” and 2 indicated “understandable and clear.” The items discussed by the expert panel were IPQ15, IPQ16, IPQ24, IPQ25, IPQ30, and C6. Overall, these did not raise any translational, linguistic, or cultural concerns. Furthermore, in order to perform the qualitative assessment of face validity (Polit and Beck 2020), a sample consisting of 50 individuals was utilized. For this purpose, they were asked to provide feedback about the difficulty of the items, the clarity of the terminology and phrases, and the relevance and suitability of the content, as well as any ambiguity associated with the wording. 300 patients diagnosed with various types of cancer were recruited face to face and by purposive sampling method from the Martyrs of Tajrish, Taleghani, and Imam Hossein hospitals in Tehran within the 9 months. The questionnaires were completed after obtaining informed consent and during outpatient appointments. In addition to completing the IPQ‐R, demographic and medical information (genotype) was also obtained. Inclusion criteria were comprised of (Rourke et al. 2007) patients aged 18–60 years with any oncological diagnosis and (Dempster et al. 2011a) receiving oncological treatment at the time of the study. Exclusion criteria were included (Rourke et al. 2007) remission of cancer and (Dempster et al. 2011a) being under palliative and terminal care. This study was approved by the ethical code (IR.SBMU.MSP.REC.1402.322) of the Research Ethics Committee, Faculty of Medicine, Shahid Beheshti University of Medical Sciences.

### 2.2 Measures

#### 2.2.1 Illness Perception Questionnaire—Revised (IPQ‐R)

Questionnaire of IPQ‐R is divided into three sections. The first section evaluates the identity subscale and presents a list consisting of 17 symptoms, in which the evaluator must indicate whether he/she considers that the symptom is or is not associated with the illness. The second section included 26 items and evaluated seven dimensions: timeline (acute/chronic), consequences, personal control, treatment control, coherence, timeline (cyclical), and emotional representation, which must be answered using a five‐point Likert scale ranging from 1 (strongly disagree) to 5 (strongly agree). The third section evaluates the causes subscale and comprises 18 items that must be answered based on the five‐point Likert scale (Moss‐Morris et al. 2002). In the original study, item selection was determined by principal components analysis (PCA) and verified the factorial structure of the questionnaire in a sample of 711 patients, including eight different illness groups (Moss‐Morris et al. 2002). Although in the original IPQ, the internal reliabilities of the control/cure and timeline dimensions were lower than those of the other dimensions, they have been improved with the inclusion of the new items in IPQ‐R, and all dimensions exhibited good internal reliability. Further analysis provided good evidence for the internal reliability of the subscales and test‐retest reliability of over a short term of a 3‐week period (0.46–0.88) and long term of a 6‐month period (> 0/5). The identity subscale demonstrated a relatively high degree of internal reliability, with a Cronbach's alpha of 0.75. All dimensions in the beliefs subscale showed good internal reliability, and the dimensions Cronbach's alpha ranged from 0.79 for the timeline cyclical to 0.89 for the timeline acute/chronic. Also, Cronbach's alpha for the causal subscale ranged from 0.86 for the psychological attributions to 0.67 for immunity (Moss‐Morris et al. 2002).

#### 2.2.2 The Positive and Negative Affect Schedule (PANAS)

The PANAS was designed to measure affect in two levels: state/trait and positive/negative. The positive affect subscale is associated with pleasurable engagement with the environment. Although the negative affect subscale reflects the dimension of general distress and assesses the variety of negative states such as anger, guilt, or anxiety. The PANAS can show relations between positive and negative affect with personality characteristics. Each subscale has 10 items rated on a five‐point Likert scale (from very low to very high). The scales have high internal consistency, are relatively uncorrelated, and are stable over a 2‐month time period (Watson et al. 1988). In Iran, the results of CFA and structural equation modeling (SEM) in a sample of 255 students with anxiety and depression disorders at the University of Tehran indicated a good fit of a two‐factor model. In this study, the validity of the two subscales was obtained 0.87 also distinguishing well between both anxiety disorders and depression (Bakhshipour and Dezhkam 2006).

#### 2.2.3 Depression, Anxiety, and Stress Scale‐21 Items (DASS)

In the current study, DASS‐21 with 21 items and three subscales (seven items for each subscale) was used. The original study reported the high reliability by calculating Cronbach's alpha coefficients for depression, anxiety, and stress as 0.91, 0.84, and 0.90, respectively. The DASS Anxiety subscale correlated with the BAI (0.81), and the DASS Depression subscale correlated with the BDI (0.74) (Lovibond 1995). In Iran, a study was conducted on 1135 nurses working in public hospitals; Cronbach's alpha coefficient was acceptable for anxiety (0.79), stress (0.91), and depression (0.93). Test‐retest reliability was also acceptable (0.740–0.881, *p* < 0.01), and the results of CFA showed acceptable fit (Kakemam et al. 2022).

#### 2.2.4 36‐Item Short‐Form Health Survey (SF‐36)

The SF‐36 questionnaire consists of eight subscales that evaluate physical function, social function, role limitation due to physical problems, role limitation due to emotional problems, mental health, vitality, bodily pain, and general health perception. Total scores on each SF‐36 subscale have ranged between 0 and 100 (Ware and Sherbourne 1992). In a study conducted in 1992 on 1007 residents (18–44 years) of Geneva in Switzerland, response rate was 82%. Completion rate for all eight dimensions of health was 95.5%. The instrument demonstrated convergent validity (100%) and discriminant validity (98%). Cronbach's alpha ranged from 0.76 to 0.92. Factorial analysis showed two principal components, corresponding to mental and physical health. Validation by independent clinical variables was also consistent with theory (Perneger et al. 1995). Reliability of a Persian translation of the SF‐36 questionnaire revealed internal consistency for eight subscales using Cronbach's alpha is 0.87. Construct validity was acceptable, and the correlation coefficient between eight subscales and related principal components was also appropriate (Motamed et al. 2005).

## 3 Statistical Analysis

Arguably we could have conducted a CFA given previous data suggesting which items are likely to display subscales. Nonetheless, given that in most previous studies all items had not been subjected to a factor analysis, we, with caution, conducted an EFA. The EFA was conducted to evaluate the factorial structure of the beliefs scale and the causal attribution scale in the Illness Perception Questionnaire. EFA was calculated using the PCA with varimax rotation. The suitability of the data for EFA was assessed by calculating the Kaiser—Meyer–Olkin (KMO) measure for sampling adequacy, where values close to one indicate high adequacy, and the acceptable threshold is above 0.60. KMO indicates the proportion of variance in the variables that might be caused by the underlying factors (Dziuban and Shirkey 1974). Further, Bartlett's Test of Sphericity was employed to evaluate the inter item correlations, which determine whether the data were suitable for factor extraction. This test must be significant at *p* < 0.05 (Backhaus et al. 2008).

We applied the factor loading cutoff points proposed by Comrey and Lee (1992), considering a minimum acceptable value of 0.32, with loadings above 0.45 deemed suitable. The optimal number of factors was determined based on parallel analysis, which is recognized as the most reliable criterion for factor retention. In parallel analysis, a random dataset with characteristics identical to the actual data are generated, and the eigenvalues of the actual data are compared with the corresponding eigenvalues from the random data. Only factors with eigenvalues greater than their corresponding random eigenvalues are retained.

The visual scree plot includes plotting the eigenvalues on a graph. It is used to establish when decreases in successive eigenvalues become less evident and is called the “elbow.” Only eigenvalues before the elbow are retained.

Internal consistency was assessed using Cronbach's alpha and McDonald's omega, which provide estimates of reliability based on the inter‐item correlations. We used McDonald's ω because it is a better alternative to Cronbach's alpha when assessing the internal consistency of short scales (Béland et al. 2017). Test‐retest reliability was also evaluated by calculating Pearson's correlation coefficient between two measurements taken 4 weeks apart. Concurrent validity was conducted utilizing Pearson's correlation coefficient to examine the relationship between the scales and related variables, ensuring that the scales demonstrate meaningful associations with related constructs. Furthermore, Pearson's correlation coefficients were calculated to examine the inter‐relationships among the subscales of the IPQ‐R. All analyses were conducted using SPSS version 27 and Mplus version 8.3.

## 4 Results

### 4.1 Characteristics of the Sample

The present study was conducted on a sample consisted of 300 cancer patients who were mostly aged 40–59 years (59%) and women (66%). Most participants were married (81%) and had at least a high school diploma or associate degree (36.7%), and only 8% were uneducated. The most common cancer diagnoses were breast cancer (34%), gastrointestinal cancer (24%), and head and neck cancer (18.3%). Regarding the duration of illness, the largest proportion of participants (38.3%) had been diagnosed within the past 7–12 months, while 27.7% were diagnosed within the last 6 months (Table 1).

**TABLE 1:** Characteristics of the study participants.

Demographic variables	*N* (%)	Disease‐related variables	*N* (%)
**Age groups**		**Diagnosis**	
20–39	51 (17)	Head and neck cancer	55 (18.3)
40–59	177 (59)	Breast cancer	102 (34)
60–79	70 (23.3)	Gastrointestinal cancer	72 (24)
Years ≥ 80	2 (0.7)	Genital cancer	40 (13.3)
**Gender**		Skeleton cancer	14 (4.7)
Women	198 (66)	Blood and lymph cancer	13 (4.3)
Men	102 (34)	Urinary tract cancer	4 (1.3)
**Education level**		**Duration of disease**	
Uneducated	24 (8)	1–6 months	83 (27.7)
Elementary school	54 (18)	7–12 months	115 (38.3)
Middle school diploma	59 (19.7)	1–2 years	47 (15.7)
High school diploma and associate degree	110 (36.7)	3–5 years	29 (9.7)
Bachelor^,^ s degree	39 (13)	Above 6 years	26 (8.7)
Master^,^ s/doctoral degree	14 (4.7)		
**Marital status**			
Married	243 (81)		
Single	33 (11)		
Divorced	10 (3.3)		
Widowed	14 (4.7)		

### 4.2 The Identity Subscale

In the first section of the study, the frequency of symptoms experienced by patients and their perceptions of whether these symptoms were relevant to their illness were examined. The most frequently reported symptom was fatigue (83.3%), followed by pain (72.7%) and loss of strength (66.3%). Next, headaches (61.3%), sleep difficulties (59.0%), weight loss (57.7%), upset stomach (57.3%), and nausea (54.3%) were reported. Other symptoms, including dizziness (51.7%), breathlessness (38.3%), stiff joints (38.3%), sore throat (32.3%), and wheeziness (29.0%), were endorsed by at least a quarter of the participants. The least frequently reported symptom was sore eyes (20.7%).

#### 4.2.1 Validity

In order to assess validity of the identity subscale, we investigated the frequencies with which different symptoms were endorsed as part of illness identity, and a similar pattern emerged. Fatigue (77.0%), pain (70.0%), and loss of strength (63.7%) were most frequently identified as illness identities. More than half of the patients also attributed nausea (52.3%) and weight loss (52.0%) to their conditions. However, symptoms such as headaches (46.3%), sleep difficulties (45.7%), dizziness (45.7%), and upset stomach (44.7%) were recognized as illness identities by fewer patients. The lowest illness identity attribution rates were observed for sore eyes (17.3%), wheeziness (23.0%), sore throat (25.7%), and stiff joints (32.0%). All the symptoms were endorsed by a percentage of the patients, indicating the validity of the range of symptoms included in the identity subscale.

#### 4.2.2 Internal Consistency

Since patients are asked first to identify symptoms they experience and then to recognize which of these symptoms they specifically associate with their illness, we in our study calculated internal consistency for two separate sections comprised of a symptom checklist and illness attribution. In this way, the internal consistency of symptom checklist and illness attribution was acceptable, with a Cronbach's alpha of 0.74 and 0.77, respectively.

### 4.3 The Beliefs Subscale

#### 4.3.1 Exploratory Factor Analysis

**FIGURE 1:**
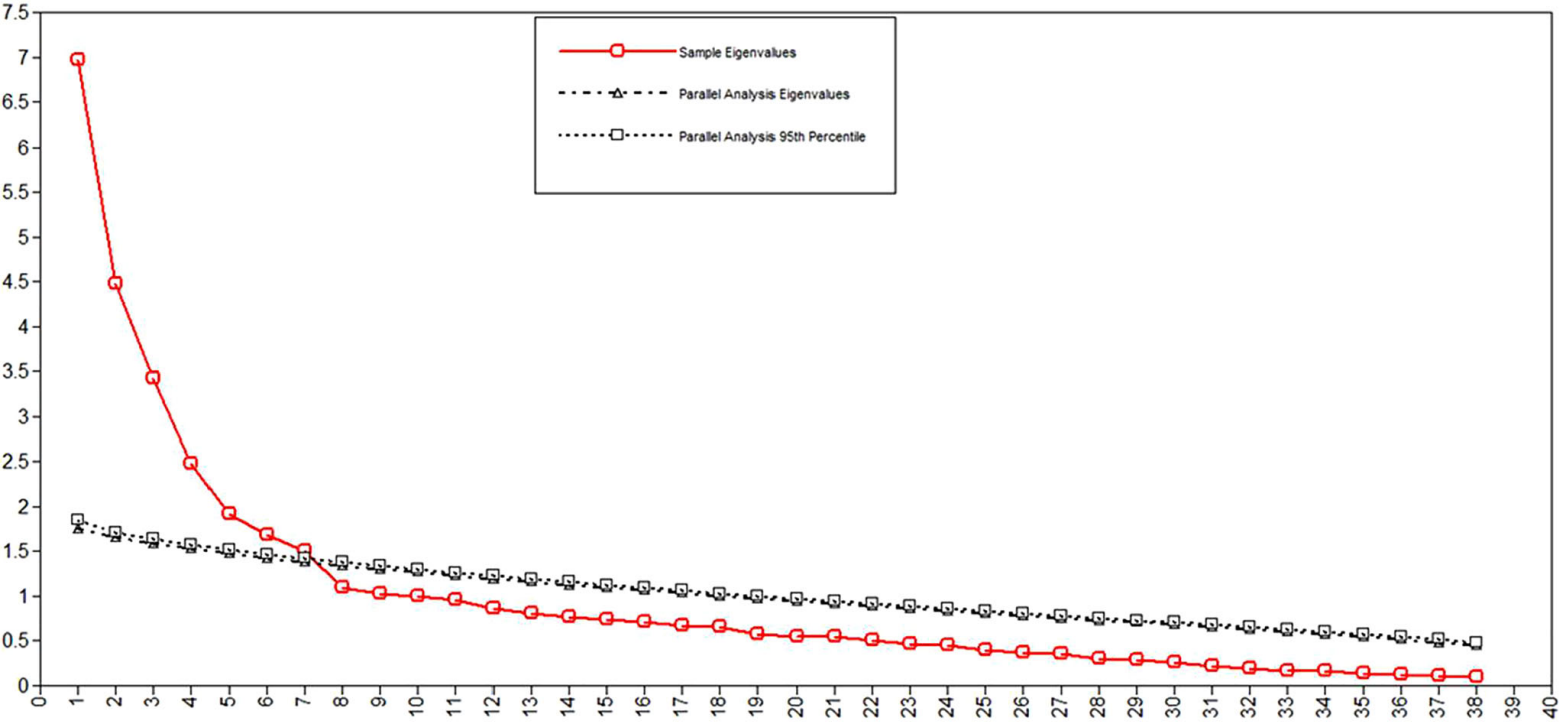
Parallel analysis of the revised illness perception questionnaire (beliefs scale).

In the second section of the questionnaire, the results of the EFA provided strong evidence for the suitability of the data to conduct this analysis. The KMO was obtained as 0.806, which exceeded the common accepted threshold of 0.60. As a result, the sample size was sufficient for reliable factor extraction. Additionally, Bartlett's Test of Sphericity revealed a statistically significant result (chi‐square = 6034.29, *p* < 0.001) and confirmed the correlation matrix is not an identity matrix and that the data are appropriate for uncovering latent factors. The parallel analysis conducted to determine the optimal number of factors suggested the retention of seven factors (Figure 1).

In whole, these seven dimensions explained 59.02% of the total variance, reflecting a meaningful and interpretable factor structure. The rotated component matrix also revealed the grouping of items into distinct dimensions and provided clear evidence of the underlying dimensions of the beliefs subscale.

The extracted factor structure closely resembled the original structure of the beliefs subscale, with only minor deviations. The items were grouped into the following factors: timeline acute/chronic (items 1, 2, 3, 4, 5, 18; 9.45% of variance), consequences (items 6, 7, 8, 9, 10, 11; 7.49% of variance), personal control (items 12, 13, 14, 15, 16, 17; 10.17% of variance), treatment control (items 20, 21, 22, 23; 8.25% of variance), illness coherence (items 24, 25, 26, 28; 6.18% of variance), timeline cyclical (items 29, 30, 31, 32; 5.74% of variance), and emotional representations (items 27, 33, 34, 35, 36, 37, 38; 11.72% of variance). The items loaded on each factor, as derived from the varimax‐rotated matrix, have been provided in Table 2.

**TABLE 2:** The revised illness perception questionnaire (beliefs scale) factor loadings.

Item	Components						
	Timeline acute/chronic	Consequences	Personal control	Treatment control	Illness Coherence	Timeline cyclical	Emotional representations
Item 1	**0.805**						
Item 2	**0.605**						
Item 3	**0.857**						
Item 4	**0.788**						
Item 5	**0.648**						
Item 6		**0.513**					
Item 7		**0.716**					
Item 8		**0.668**					
Item 9		**0.451**					
Item 10		**0.478**					
Item 11		**0.615**					
Item 12			**0.397**			0.452	
Item 13			**0.846**				
Item 14			**0.802**				
Item 15			**0.809**				
Item 16			**0.827**				
Item 17			**0.801**				
Item 18	**0.558**			−0.359			
Item 19		−0.438				0.365	
Item 20				**0.903**			
Item 21				**0.896**			
Item 22				**0.914**			
Item 23				**0.556**			
Item 24					**0.471**		
Item 25					**0.714**		
Item 26					**0.854**		
Item 27							**0.570**
Item 28					**0.750**		
Item 29						**0.638**	
Item 30						**0.625**	
Item 31						**0.435**	
Item 32						**0.644**	
Item 33							**0.807**
Item 34							**0.828**
Item 35							**0.673**
Item 36							**0.689**
Item 37							**0.826**
Item 38							**0.847**
Eigenvalues	3.59	2.85	3.86	3.13	2.35	2.18	4.45
% of variance	9.45	7.49	10.17	8.25	6.18	5.74	11.72
Cronbach's alpha	0.842	0.706	0.863	0.878	0.716	0.620	0.890
McDonald's omega	0.865	0.719	0.870	0.894	0.738	0.627	0.889

*Note*: Bold values indicate factor loadings = 0.32.

Notable changes in the factor structure included the following items: Item 27 (“My illness does not make any sense to me”), which had been loaded on illness coherence in the original study, in our study was loaded on emotional representations. Item 19 (“There is very little that can be done to improve my illness”) demonstrated cross‐loading, whose its dominant loading was negatively associated with consequences; therefore, it was excluded from the factor structure. Moreover, item 12 (“There is a lot that I can do to control my symptoms”) indicated cross‐loading between personal control and timeline cyclical but retained its original dimension (personal control), corresponding to the original study.

#### 4.3.2 Concurrent Validity

The concurrent validity of the IPQ‐R (beliefs subscale) was assessed by examining the bivariate correlations with DASS (depression, anxiety, stress), PANAS (positive and negative affect), and SF‐36 Health Survey. There was significant correlation between most seven dimensions of the IPQ‐R and the other measures. A negative correlation found between timeline acute/chronic and PANAS (positive symptoms), also positive correlations with DASS (depression, anxiety), SF‐36, and PANAS (negative symptoms) revealed. Consequences demonstrated positive correlations with DASS (depression, anxiety, stress), PANAS (negative symptoms) and SF‐36; moreover, they had a negative correlation with PANAS (positive symptoms). Personal control negatively associated with DASS (depression and anxiety) and SF‐36 but was positively correlated with PANAS (positive symptoms). There were negative relationships between treatment control and DASS (Depression) and PANAS (negative symptoms); in addition a positive relationship was obtained with PANAS (positive symptoms). Illness coherence indicated negative correlations with DASS (depression, anxiety, stress) and PANAS (negative symptoms) but had a positive correlation with PANAS (positive symptoms). The positive correlations observed among timeline cyclical with DASS (depression, anxiety, stress), PANAS (negative symptoms) and SF‐36, while they showed a negative correlation with PANAS (positive symptoms). Emotional representations demonstrated positive correlations with DASS (depression, anxiety, stress), PANAS (negative symptoms), and SF‐36, versus showing a positive correlation with PANAS (positive symptoms). Overall, all correlations were statistically significant both at the 0.01 and 0.05 levels (Table 3).

**TABLE 3:** Bivariate correlations between DASS, PANAS, and SF‐36 health survey with the the revised illness perception questionnaire (beliefs subscale).

	DASS depression	DASS anxiety	DASS stress	PANAS positive	PANAS negative	SF‐36 health survey
Timeline acute/chronic	0.143^*^	0.121^*^	0.096	−0.184^**^	0.181^**^	0.186^**^
Consequences	0.320^**^	0.246^**^	0.340^**^	−0.287^**^	0.368^**^	0.426^**^
Personal control	−0.175^**^	−0.126^*^	−0.099	0.223^**^	−0.108	−0.162^**^
Treatment control	−0.123^*^	0.095	0.075	0.215^**^	−0.127^*^	0.035
Illness coherence	−0.223^**^	−0.166^**^	−0.197^**^	0.166^**^	−0.146^*^	−0.103
Timeline cyclical	0.317^**^	0.290^**^	0.279^**^	−0.243^**^	0.283^**^	0.339^**^
Emotional representations	0.371^**^	0.428^**^	0.499^**^	−0.293^**^	0.567^**^	0.409^**^

**p < 0.01 and *p < 0.05.

#### 4.3.3 Internal Consistency

The internal consistency of the IPQ‐R was computed using Cronbach's alpha and McDonald's omega coefficients for the beliefs subscale and its dimensions. Cronbach's alpha coefficients were as follows: total scale (0.790), timeline acute/chronic (0.842), Consequences (0.706), personal control (0.863), treatment control (0.878), illness coherence (0.716), timeline cyclical (0.620), and Emotional representations (0.890). These values indicated good to excellent internal consistency for most dimensions, with the exception of the timeline cyclical, which reflected moderate consistency.

Similarly, McDonald's omega coefficients were obtained for the total subscale (0.715), timeline acute/chronic (0.865), Consequences (0.719), personal control (0.870), treatment control (0.894), illness coherence (0.738), timeline cyclical (0.627), and Emotional representations (0.889). The omega values as well as the reliability of the subscale and its dimensions were confirmed with slight variations compared to the alpha coefficients.

#### 4.3.4 Test‐Retest Reliability

To assess the test‐retest reliability of the IPQ‐R (beliefs subscale), it was administered to 50 patients on two occasions with a 4‐week interval. Pearson correlation coefficients were calculated to examine the stability of responses over time. The results indicated high test‐retest reliability in all dimensions, with correlation coefficients ranging from 0.775 to 0.931 (all *p* < 0.01). Emotional representations showed the highest stability (*r* = 0.931), reflecting excellent agreement between the T1 and T2. Afterward, both timeline acute/chronic dimensions (*r* = 0.887) and consequences (*r* = 0.871) demonstrated strong reliability. timeline cyclical (*r* = 0.868) and illness coherence (*r* = 0.855) also revealed high stability over time. Moreover, personal control (*r* = 0.787) and treatment control (*r* = 0.775) demonstrated satisfactory reliability, reflecting consistent responses across the two testing occasions. These findings provide evidence for the excellent reliability of the instrument beliefs subscale of the IPQ‐R in a sample of cancer patients.

### 4.4 The Causal Attribution Subscale

#### 4.4.1 Exploratory Factor Analysis

**FIGURE 2:**
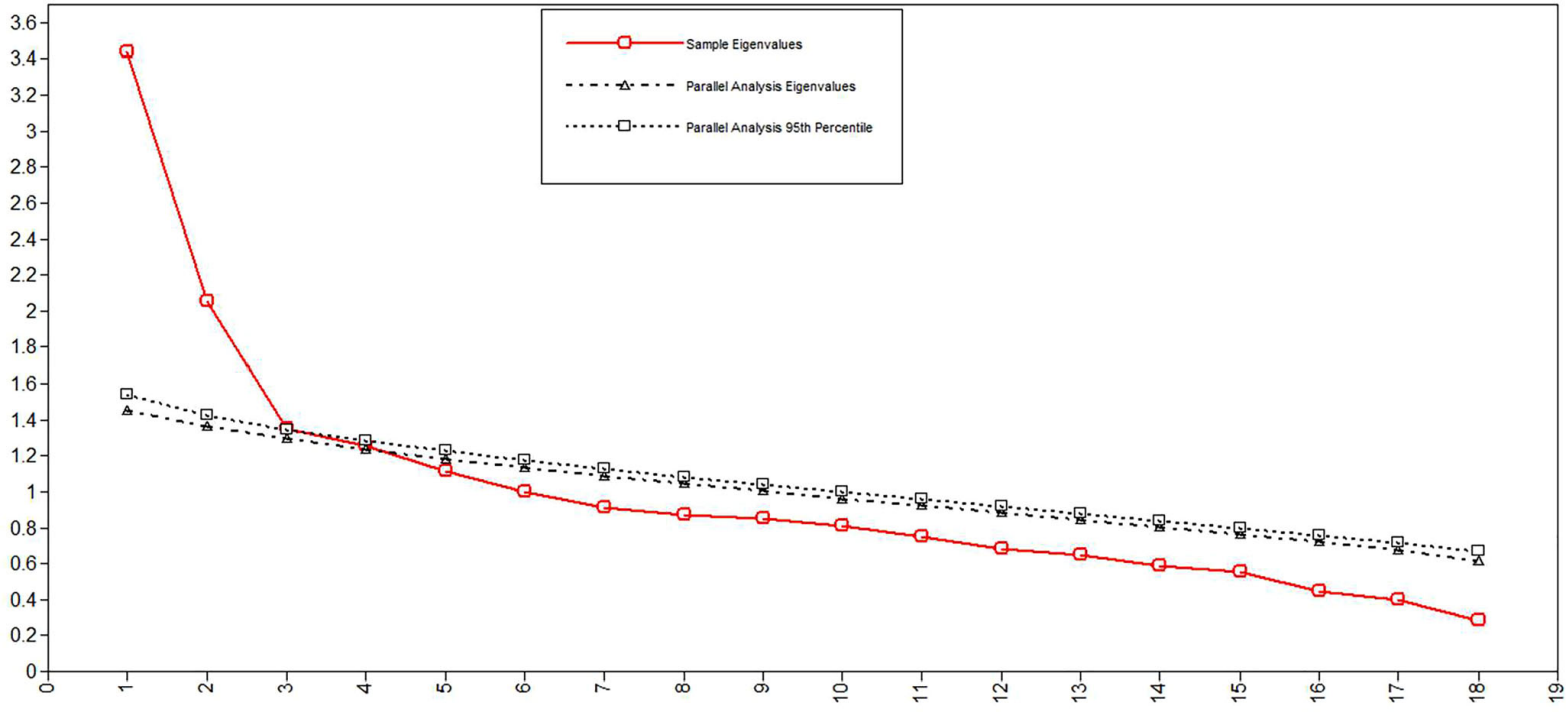
Parallel analysis of the revised illness perception questionnaire (causal attribution scale).

The EFA for the causal attribution subscale of the questionnaire identified a three‐factor structure based on parallel analysis criteria. The parallel analysis scree plot (Figure 2) supported the retention of three factors, which account for the underlying structure of the causal attribution scale. Compared to the original study, the two components of smoking and alcohol consumption shared their items, suggesting they refer to a single factor. This factor was labeled substance use. The KMO was 0.754, indicating sufficient data for factor analysis. Bartlett's Test of Sphericity was significant (*χ* ^2^ = 942.41, *p* < 0.001) and confirmed the suitability of the data for factor extraction.

Psychological attributions include items 1, 8, 9, 10, 12, and 17, which had an eigenvalue of 3.38 and explained 18.76% of the variance. In this factor, items 2, 4, 5, and 18 were excluded due to low factor loadings. Risk factors contain items 3, 6, 7, 11, 13, and 16, which had an eigenvalue of 1.73 and explained 9.63% of the variance. Substance use with items 14 and 15 also revealed an eigenvalue of 1.73 and accounted for 9.61% of the variance. The items loaded on each factor, as derived from the varimax‐rotated matrix, have been provided in Table 4.

**TABLE 4:** The revised illness perception questionnaire (causal attribution subscale) factor loadings.

Items	Components		
	Psychological attributions	Risk factors	Substance use
Item 1	**0.680**		
Item 2			
Item 3		**0.512**	
Item 4			
Item 5			
Item 6		**0.413**	
Item 7		**0.556**	
Item 8	**0.644**		
Item 9	**0.666**		
Item 10	**0.778**		
Item 11	0.312	**0.494**	
Item 12	**0.807**		
Item 13		**0.593**	
Item 14			**0.852**
Item 15			**0.829**
Item 16		**0.445**	
Item 17	**0.649**		
Item 18			
Eigenvalues	3.38	1.73	1.73
% of variance	18.76	9.63	9.61
Cronbach's alpha	0.808	0.452	0.693
McDonald's omega	0.813	0.456	—

#### 4.4.2 Concurrent Validity

The concurrent validity of the causal attribution subscale was evaluated by examining the correlations of its three factors (psychological attributions, risk factors, and substance use) with DASS (depression, anxiety, stress), PANAS (positive and negative Affect), and the SF‐36 Health Survey. Psychological attributions revealed strong positive correlations with DASS (depression, anxiety, stress), PANAS (negative symptoms), and SF‐36, but no significant correlation was observed with PANAS (positive symptoms). Risk factors exhibited positive correlations with DASS (depression, anxiety, stress), PANAS (negative symptoms), and SF‐36, while no significant correlation was found with PANAS (positive symptoms). Substance use did not show any significant correlations with DASS, PANAS, or the SF‐36 Health Survey, reflecting limited concurrent validity for this factor in relation to other factors. These findings express that the psychological attributions have stronger association with emotional distress and health indicators. Although risk factors displayed weaker correlation, substance use did not correlate significantly with other calculated indicators (Table 5).

**TABLE 5:** Bivariate correlations between DASS, PANAS, and SF‐36 health survey with the the revised illness perception questionnaire (causal attribution scale).

	DASS depression	DASS anxiety	DASS stress	PANAS positive	PANA negative	SF‐36 health survey
Psychological attributions	0.292^**^	0.322^**^	0.398^**^	−0.120	0.438^**^	0.352^**^
Risk factors	0.162^**^	0.194^**^	0.198^**^	−0.037	0.125^*^	0.127^*^
Substance use	−0.098	−0.060	−0.052	0.036	0.010	−0.061

**p < 0.01 and *p < 0.05.

#### 4.4.3 Internal Consistency

The psychological attributions factor demonstrated good internal consistency, with Cronbach's alpha of 0.808 and McDonald's omega of 0.813. The risk factors showed low reliability, with Cronbach's alpha of 0.452 and McDonald's omega of 0.456. Also, the substance use factor had moderate Cronbach's alpha of 0.693. In the case of McDonald's omega, it could not be computed for this factor due to insufficient items.

#### 4.4.4 Test‐Retest Reliability

The test‐retest reliability of IPQ‐R (causal attribution subscale) was examined over a 4‐week interval in a sample of 50 patients. The results demonstrated a moderate to high test‐retest reliability across the factors, which was in the range from (0.768) to (0.917). Psychological attributions indicated the highest stability (*r* = 0.917, *p* < 0.01), reflecting excellent consistency over time. Afterward, both the substance use (*r* = 0.779, *p* < 0.05) and risk factor (*r* = 0.768, *p* < 0.01) were calculated, displaying satisfactory reliability.

### 4.5 The Inter‐Correlations Among IPQ‐R Dimensions

Pearson correlation coefficients were calculated to examine the inter‐relationships among the subscales of the IPQ‐R (see Table 6). The identity subscale showed significant positive correlations with timeline acute/chronic, consequences, timeline cyclical, and emotional representations. Nonetheless, unlike the original study, no relationship was found among it, personal control, treatment control, and illness coherence. Meanwhile, timeline acute/chronic, consequences, timeline cyclical, and emotional representations had a positive relationship with each other. Although consistent with the original study, personal control, treatment control, and illness coherence indicated a positive relationship with each other. Further, personal control, treatment control, and illness coherence were negatively associated with timeline acute/chronic, consequences, timeline cyclical, and Emotional representations. In the third subscale (causal attribution), substance use with timeline acute/chronic, illness coherence, and Emotional representations revealed negative associations. Risk factors with treatment control and illness coherence had negative correlations, and versus the timeline cyclical had positive correlations. Psychological attributions were positively associated with consequences, timeline cyclical and emotional representations. Moreover, between psychological attributions and risk factors and also between risk factors and substance use, there were positive relationships. But no relationship was found between (psychological attributions) and (substance use). In the same way, identity subscale exhibited a positive correlation with psychological attributions but showed no relationship with risk factors and substance use.

**TABLE 6:** Correlation matrix of the IPQ‐R dimensions.

	1	2	3	4	5	6	7	8	9	10	11
1. Identity	—										
2.Timeline acute/chronic	0.139^*^	—									
3.Consequences	0.330^**^	0.333^**^	—								
4.Personal control	0.001	−0.263^**^	−0.114^*^	—							
5.Treatment control	0.031	−0.225^**^	−0.019	0.324^**^	—						
6.Illness coherence	−0.004	0.055	0.001	0.159^**^	0.177^**^	—					
7.Timeline cyclical	0.292^**^	0.197^**^	0.283^**^	−0.041	−0.194^**^	−0.207^**^	—				
8.Emotional representations	0.316^**^	0.177^**^	0.442^**^	−0.065	−0.123^**^	−0.199^**^	0.329^**^	—			
9.Psychological attributions	0.244^**^	−0.014	0.190^**^	0.044	0.105	0.062	0.135^*^	0.366^**^	—		
10.Risk factors	0.080	−0.036	0.071	0.000	−0.170^**^	−0.143^*^	0.213^**^	0.081	0.118^*^	—	
11.Substance use	−0.061	−0.120^*^	0.068	−0.070	−0.050	−0.114^*^	0.029	−0.128^*^	0.081	0.138^*^	—

**p < 0.01; *p < 0.05.

## 5 Discussion

As it was noted in the introduction, the IPQ‐R has strengthened the psychometric properties of the original scale (IPQ) in a number of ways, including improving the reliability of the subscales. This instrument is based on Leventhal's self‐regulation theory and proposes how individuals construct schematic representations of their illness according to the concrete and abstract of information. When an individual confronts a trauma (such as cancer), existing cognitive schemas are activated, and cognitive processing, may give a new meaning to his/her illness. In this state, the activating of cognitive processing prompts a person to revise their assumptions and give a new meaning to their illness (Ogińska‐Bulik 2016). As both medical treatment adherence and psychotherapy are grounded in a cultural context and must be congruent with the patient's cultural beliefs of his or her illness in order to be effective, attention to cultural and cross‐cultural differences in illness representations becomes increasingly important (Leventhal et al. 2020). This paper aimed to further examine the psychometric properties of the IPQ‐R in a cancer population, whose results have been explained below.

First, we explain validity and reliability of the identity subscale of the first section in relation to cancer. To determine validity, we asked patients first to identify symptoms they experience and then to determine which of these symptoms specifically as part of illness designate association with their illness. Indeed, the identity subscale reflects the illness label. The results of validation were analogous to the results obtained by Moss‐Morris et al. (2002), and the symptoms, which are often associated with the illness (regardless of its type), were fatigue and pain, respectively. According to Leventhal's self‐regulatory model, the perception of symptoms that are consisted of the illness identity subscale is the result of the interaction of symptoms and social messages. Therefore, the further frequency of fatigue and pain symptoms could be linked to the messages of caregivers and treatment staff to cancer patients, besides the experienced symptoms. These findings highlight variability in symptom perception, that being widely recognized as part of the illness identity. Together, all the symptoms were endorsed by a percentage of the patients, confirming validity of the range of symptoms included in the identity subscale.

Because the identity subscale consists of distinct symptoms and some of these symptoms can be more relevant to particular illnesses than others, internal consistency of this subscale is less than that of the other subscales (Moss‐Morris et al. 2002). Nevertheless, our study demonstrated a relatively high degree of internal reliability, with a Cronbach's alpha of 0.74 and 0.77 for symptom checklist and illness attribution, respectively. As we expected, due to that in our study the analyses were conducted on the responses of symptom checklist and illness attribution separately, their reliability improved.

The EFA was conducted to validate *the second section* and provided the seven dimensions, closely resembled the original structure of the beliefs subscale. In following we explain some of the changes: Item 27 (“My illness does not make any sense to me”), which had appeared on the illness coherence dimension in the original study, in our research was loaded on the Emotional representations dimension. The illness coherence (how the illness “makes sense” as a whole) can be thought of as a type of meta‐cognition reflecting the way in which the patient has provided a coherent understanding of the illness and may play an important role in longer term adjustment (Moss‐Morris et al. 2002). Thus, it would be logical to move item 27 from this factor to the emotional representations factor. Because the emotional representation construct has strong relationships with a cognitive representation construct. A finding that is in keeping with Leventhal et al.’s model is that there may be conceptual overlap among the cognitive and emotional components of the model. In addition, this finding may partly reflect a semantic issue in the Persian translation of the item. While the original item primarily refers to cognitive understanding and meaning‐making, the Persian wording was closer to “This illness does not create any feeling in me,” which may evoke emotional rather than cognitive interpretations for Iranian respondents. As a result, the item may have been perceived more as an emotional experience than a question of illness coherence, contributing to its loading on the emotional representations factor. Item 12 (“There is a lot that I can do to control my symptoms”) indicated cross‐loading between the personal control and timeline cyclical dimensions. Indeed, the cross‐loading may reflect some overlap between symptom controllability and fluctuations in symptom experience among patients with cancer. But retained within its original dimension (personal control), corresponding to the original study. Based on the content of the item, obviously, this choice was conceptually appropriate. Item 19 (“There is very little that can be done to improve my illness”), originally belonging to the treatment control dimension, demonstrated substantial cross‐loading and showed a stronger negative loading on the Consequences factor. This finding may suggest that, within Iranian patients with cancer, beliefs regarding limited treatment effectiveness are not experienced solely as reduced treatment control but may also reflect broader perceptions of illness severity, hopelessness, and anticipated negative consequences. It was already mentioned in the introduction that the illness perception among cancer patients is related to their cultural background (Dein 2004). For instance, item 19 is difficult to understand and ambiguous in different cultural contexts. In Iranian culture, patients often adapt to health threats by adopting strategies such as “letting fate take its course,” believing conflict with fate will lead to correspondingly undesirable outcomes.

In the present study, among personal control, treatment control, and illness coherence with positive affect dimension (PANAS) had a positive relationship. On the other hand, these dimensions correlated negatively with negative affect dimension, DASS, and SF‐6. Since the items in personal control, treatment control, and illness coherence refer to the patient's positive belief who is be able to control his or her symptoms, the existence of these relationships is sensible. In contrast, the Consequences, Timeline, Cyclical timeline, and Emotional representation revealed negative relationships with the positive affect dimension (PANAS) and, on the contrary, positive relationships with negative affect dimension, DASS, and SF‐6. Because items in these dimensions evaluate subjective distress and discomfort, this result is not surprising and thus will not be further discussed.

Generally all the dimensions of the beliefs subscale demonstrated good internal consistency. Cronbach's alpha and McDonald's omega for the total scale were characterized by a satisfactory level of measurement reliability 0.79 and 0.71, respectively. Only dimension of the timeline cyclical revealed moderate consistency for both Cronbach's alpha and McDonald's omega, 0.620 and 0.670, respectively. In the original study also, timeline cyclical had lower reliability than other dimensions. Because the timeline is cyclical and implies to the duration and course of illness, this is not surprising; variable responses to symptoms can be given in different disease cycles. The results also indicated high test‐retest reliability in all dimensions, and correlation coefficients were ranging from 0.775 to 0.931 (all *p* < 0.01). In particular, emotional representations showed the highest stability (*r* = 0.931), reflecting excellent agreement between the T1 and T2. According to Leventhal's model, emotional representations lead to emotional coping, which is different for each person and is characterized by the choice of various emotional strategies such as anxiety, depression, anger, etc. The higher test‐retest score on this dimension indicates that emotional responses are more stable over time than other dimensions. We can argue that this is due to the positive and significant relationship between emotional representations and psychological attributions. Since the psychological attributions of a cognitive construct are relatively stable, emotions associated with it are also more stable. The implied meaning of this sentence is that further training is needed to modify emotional responses when individuals are exposed to a trauma like cancer. In the initial study, patients who had more psychological attributions considered their illness as chronic and experienced more emotional distress (Moss‐Morris et al. 2002). The authors also highlighted the 1 month test‐retest reliability coefficients in patients undergoing treatment, as well as patients with chronic diseases, are particularly high. In contrast, the 3 and 6 month test‐retest correlation coefficients were progressively lower, and this is not surprising since self‐regulation theory asserts that illness representations may change over time. This is especially likely if the illness is unstable or if the profile of the illness changes (Moss‐Morris et al. 2002). However, our study sample domesticated that regardless of cancer type, treatment process and time are more important in the consistency of results.

The factorial solution of *the third section* provided the three factors. These factors are strongly characterized by psychological and medical variables along with pathogenic and therapeutic properties, which is particularly important in the case of cancer (Meder and Didkowska 2011). Compared to the original study, in our study the two factors of smoking and alcohol consumption shared their items and comprised a factor (substance use). This overlap is conceptually comprehensible because both smoking and alcohol use factors are part of the more general factor of substance use. Again, given that the variety of questions in these two factors is low (one item each), it is likely that two related factors have merged. This solution is also recommended by the tool's original authors (Weinman et al. 1996; Moss‐Morris et al. 2002) when it is impossible to isolate reliable subscales within the Causes subscale.

Psychological attributions and risk factors with DASS, SF‐36, and the negative affect dimension (PANAS) demonstrated significant positive relationships, which was expected given the negative content of both the factors' items. In contrast, there were no significant relationships among substance use, DASS, SF‐36, and PANAS. We continue to believe this is important because of the powerful influence unique characteristics of an illness and particular cultural factors can play in understanding patients’ perceptions. For instance, “God's will.” It should be noted, in the Iranian socio‐cultural context, substance use (particularly alcohol consumption) remains a socially sensitive and stigmatized issue, which may influence endorsement patterns and reduce variability in responses.

In this study, the results of computing Cronbach's alpha and McDonald's omega in psychological attributions and the risk factors obtained good (0.80 and 0.81) and low (0.45 and 0.45), respectively. We can hypothesis that low internal consistency of the risk factor may be more likely due to the heterogeneity of its items. On the other hand, based on the initial study (Weinman et al. 1996), the Causal attribution subscale depends on the type of illness and strongly characterized by individual psychological and medical variables describing the illness and treatment, which is particularly important in the case of cancer (Meder and Didkowska 2011). Because they may simultaneously endorse biomedical, psychological, environmental, and fatalistic explanations for illness causation. Likewise, Cronbach's alpha in the substance use factor was 0.69 that is a moderate reliability. The substance use factor consisted of only two items, which may partly explain its low reliability coefficient. As well, psychological attributions (from the causal attribution subscale) with test‐retest (*r* = 0.917, *p* < 0.01) appeared to be most consistent, like patients emotional representations. Given that psychological attributions are cognitive constructs and stable, mostly indicative of a style of thinking, resistance to change over time is obvious.

The revised IPQ‐R dimensions appear to show logical inter‐relationships. Like the original study, in our study also beliefs in treatment and personal control and illness coherence (dimensions with positive content) were inversely related to pessimistic beliefs in the timeline acute/chronic, consequences, timeline cyclical of the illness and negative emotional representations (dimensions with negative content). The positive relationship between the personal control and treatment control may be caused by the fact that these dimensions, in line with Leventhal's model, formed one dimension in the original version of the IPQ and, again, like two timelines, acute/chronic and timeline cyclical (Leventhal et al. 2020). Also, this may reflect the fact that the distinction will be less important in some illnesses than in others. For instance, in MS, where no specific treatment may be prescribed, patients may view treatment choices and should not see it as a personal choice, because the condition for decision‐making is more complicated. On the other hand, in illnesses such as cancer, where medical treatment is very prescriptive, choosing and controlling the treatment path is more predictable (Reichardt et al. 2018). In the same vein, the existence of positive correlations timeline acute/chronic, consequences, timeline cyclical, and emotional representations with the identity subscale are rational because of their negative content. Meanwhile, non‐relationship among personal control, treatment control, and illness coherence with the identity subscale are also rational. Probably, negative relationships between timeline acute/chronic and substance use are due to the fear of substance dependence, which has been considered in the perception of both chronic and acute states of disease. Analogously with the original study, consequences and emotional representations were positively associated with psychological attributions. It is clear when patients attribute their illness to psychological causes, perceive more symptoms and more serious consequences, and also experience more emotional distress. The negative relationship between emotional representations and substance use may be because substance use is associated with unpleasant emotional experiences. In the case of positive relationships among timeline that are cyclical with psychological attributions and risk factors, it is likely that fluctuating nature of disease‐related perceptions over time will be relevant to the instability of attributions and risk factors. The theory by Antonovsky (2002) and research based on this theory, for example, Piotrowicz and Cianciara (2011), have confirmed the existence of positive relationships among the sense of coherence and positive health indicators (e.g., optimism, self‐esteem, and quality of life). In this way, the negative relationships among illness coherence, risk factors, and substance use are logical. This suggests that people fail to make sense of their illness (illness coherence) if they endorse behavioral and psychological causal factors such as smoking, diet, alcohol, drugs, stress, or overwork. Unlike the original study, in our study personal control did not have significant relationship with any of the factors of the third subscale (causal attribution). On theoretical grounds, it seems logical that personal control with positive content has no relationship with any of the factors of causal attribution subscale with negative content. On the other hand, because causes that could be perceived as triggering or maintaining an illness are specific to the illness and culture, the results of the present study are not surprising (Oudin Doglioni et al. 2021). Unlike the initial study, existence of a negative correlation between treatment control and risk factors suggests that patients feel less control over their illness treatment when they confirm behavioral and psychological factors such as attributions, smoking, diet, alcohol, stress, or overwork. The positive relationship between psychological attributions and risk factors could be because patients, when they attribute their illness to psychological causes, also consider the physical factors of their illness, which have been incorporated in the content of the risk factor. As we expected, risk factors had a positive relationship with substance use, probably because the perception of the cause of the disease is present in the content of the items of both factors. On the other hand, no significant correlation was found between psychological attributions and substance use, which could be because attributions are characterized by psychological and medical variables, while the other is not. There were positive relationships between the illness identity subscale and psychological attributions factor. This is congruent with previous findings, which demonstrate patients with more severe symptoms report a greater number of illness attributions (Affleck et al. 1987). In other words, patients who attribute their illness to psychological factors perceive and report more symptoms.

## 6 Conclusion

Although relationships among these constructs suggest that the emotional representations have relationships with cognitive representations, which is in line with Leventhal's conceptual model. But the correlations among these constructs have significantly different from unity. Using a validated a tool like IPQ‐R, future research can examine how illness representation would influence treatment‐seeking behaviors and remission. In other words, beyond its psychometric properties, the Persian version of the IPQ‐R may also have important clinical utility in oncology settings. Assessing illness perceptions in patients with cancer can help clinicians identify maladaptive beliefs related to hopelessness, catastrophic interpretations of the illness, low perceived control, and emotional distress. Given the strong cultural and emotional meanings associated with cancer in the Iranian context, the instrument may additionally facilitate more effective clinician–patient communication and support psychologically informed interventions, including psycho‐education and supportive psychological care. The IPQ‐R may therefore serve as a useful tool for both clinical assessment and psycho‐oncology research in Iranian cancer populations.

## 7 Limitations and Suggestions

One of the strengths of the present study is that the psychometric properties of all three sections of the IPQ‐R have been calculated and reported. The second strength of the study is that we recruited patients in the active phase of the disease because the studies have indicated that statistically significant differences exist between illness representations in the active and remission phases of the disease (Vegni et al. 2019). Because illness perception is a mental construct that can be changed, it might be possible for cancer patients to adopt adaptive coping strategies (cognitive, emotional, and behavioral) through intervention in the way they perceive their illness (Petrie et al. 2002; Moss‐Morris et al. 2007). This may significantly help the improvement of the patient's health, their quality of life, and as well as the optimization of the doctor–patient or therapist–patient relationship. However, the evidence presented in this study is exploratory, so it is therefore suggested that subsequent studies confirm IPQ‐R structural dimensionality by CFA in cancer patients. A further limitation of the present study was the unequal distribution of cancer types within the sample, with some diagnoses being overrepresented and others absent. Since illness perceptions may vary across different cancer conditions, the generalizability of the findings to all cancer populations should be interpreted with caution. And again, compared with the Beliefs subscale, the Causal Attribution subscale demonstrated weaker psychometric performance. Although a meaningful three‐factor structure was identified, some dimensions showed low reliability and limited evidence of concurrent validity. These findings suggest that the causal attribution component of the IPQ‐R may be more sensitive to cultural and contextual influences and should therefore be interpreted with caution in Iranian patients with cancer. Further refinement and evaluation of this subscale may be warranted in future studies.

## Author Contributions

**Maryam Amini Fasakhoudi**: conceptualization, writing – original draft, writing – review and editing. **Mohammad Noori**: project administration. **Hossein Karsazi**: writing – original draft, software, methodology. **Asma Shahi**: writing – review and editing. **Narin Beyraghdar**: data curation.

## Ethics Statement

Ethics approval and consent to participate in this study were approved by the ethical code (IR.SBMU.MSP.REC.1402.322) of School of Medicine, Shahid Beheshti University of Medical Sciences. All procedures performed in studies involving human participants were in accordance with the ethical standards of the institutional and/or national research committee and with the 1964 Helsinki Declaration and its later amendments or comparable ethical standards.

## Funding

The authors have nothing to report.

## Conflicts of Interest

The authors declare no conflicts of interest.

## Data Availability

The data that support the findings of this study are available from the corresponding author upon reasonable request.

## References

[brb371603-bib-0001] Affleck, G. , H. Tennen , S. Croog , and S. Levine . 1987. “Causal Attribution, Perceived Control, and Recovery from a Heart Attack.” Journal of Social and Clinical Psychology 5, no. 3: 339–355. 10.1521/jscp.1987.5.3.339. 3571655

[brb371603-bib-0002] Antonovsky, A. 2002. “Unraveling the Mystery of Health: How People Manage Stress and Stay Well.” In The Health Psychology Reader 127–139. SAGE Publications Ltd.

[brb371603-bib-0003] Ashley, L. , A. B. Smith , A. Keding , H. Jones , G. Velikova , and P. Wright . 2013. “Psychometric Evaluation of the Revised Illness Perception Questionnaire (IPQ‐R) in Cancer Patients: Confirmatory Factor Analysis and Rasch Analysis.” Journal of Psychosomatic Research 75, no. 6: 556–562. 10.1016/j.jpsychores.2013.08.005. 24290046

[brb371603-bib-0004] Backhaus, K. , B. Erichson , W. Plinke , and R. Weiber . 2008. Multivariate Analysemethoden. Springer‐Verlag.

[brb371603-bib-0005] Bakhshipour, R. , and M. Dezhkam . 2006. “A Confirmatory Factor Analysis of the Positive Affect and Negative Affect Scales (PANAS).” Journal of Psychology 9, no. 4: 351–365.

[brb371603-bib-0006] Béland, S. , D. Cousineau , and N. Loye . 2017. “Utiliser Le Coefficient Omega De McDonald À la Place De L'alpha De Cronbach.” McGill Journal of Education 52, no. 3: 791–804.

[brb371603-bib-0007] Chen, J. , H. Zhang , R. Suo , et al. 2020. “Adaptation and Psychometric Testing of the Chinese Version of the Revised Illness Perception Questionnaire for Cervical Cancer Patients.” European Journal of Oncology Nursing 48: 101799. 10.1016/j.ejon.2020.101799. 32750660

[brb371603-bib-0008] Comrey, A. L. , and H. B. Lee . 1992. A First Course in Factor Analysis 2nd ed. Lawrence Erlbaum Associates.

[brb371603-bib-0009] de Rooij, B. H. , M. S. Thong , J. van Roij , C. S. Bonhof , O. Husson , and N. P. Ezendam . 2018. “Optimistic, Realistic, and Pessimistic Illness Perceptions; Quality of Life; and Survival Among 2457 Cancer Survivors: The Population‐Based PROFILES Registry.” Cancer 124, no. 17: 3609–3617. 30192384 10.1002/cncr.31634

[brb371603-bib-0010] Dein, S. 2004. “Explanatory Models of and Attitudes towards Cancer in Different Cultures.” Lancet Oncology 5, no. 2: 119–124. 10.1016/S1470-2045(04)01386-5. 14761816

[brb371603-bib-0011] Dempster, M. , and N. K. McCorry . 2012. “The Factor Structure of the Revised Illness Perception Questionnaire in a Population of Oesophageal Cancer Survivors.” Psycho‐Oncology 21, no. 5: 524–530. 10.1002/pon.1927. 21308860

[brb371603-bib-0012] Dempster, M. , N. K. McCorry , E. Brennan , M. Donnelly , L. Murray , and B. T. Johnston . 2012. “Psychological Distress among Survivors of Esophageal Cancer: The Role of Illness Cognitions and Coping.” Diseases of the Esophagus 25, no. 3: 222–227. 10.1111/j.1442-2050.2011.01233.x. 21819485

[brb371603-bib-0013] Dempster, M. , N. K. McCorry , E. Brennan , M. Donnelly , L. J. Murray , and B. T. Johnston . 2011a. “Illness Perceptions among Carer–Survivor Dyads are Related to Psychological Distress Among Oesophageal Cancer Survivors.” Journal of Psychosomatic Research 70, no. 5: 432–439. 10.1016/j.jpsychores.2010.07.007. 21511073

[brb371603-bib-0014] Dempster, M. , N. K. McCorry , E. Brennan , M. Donnelly , L. J. Murray , and B. T. Johnston . 2011b. “Do Changes in Illness Perceptions Predict Changes in Psychological Distress Among Oesophageal Cancer Survivors?.” Journal of Health Psychology 16, no. 3: 500–509. 10.1177/1359105310386633. 21224333

[brb371603-bib-0015] Oudin Doglioni, D. , A.‐L. Pham‐Hung D'Alexandry D'Orengiani , F. Galactéros , and M.‐C. Gay . 2021. “Psychometric Characteristics of the Revised Illness Perception Questionnaire (IPQ‐R) in Adults with Sickle Cell Disease.” Health Psychology and Behavioral Medicine 10, no. 1: 60–80. 10.1080/21642850.2021.2016411. 34993006 PMC8725854

[brb371603-bib-0016] Dziuban, C. D. , and E. C. Shirkey . 1974. “When is a Correlation Matrix Appropriate for Factor Analysis? Some Decision Rules.” Psychological Bulletin 81, no. 6: 358–361. 10.1037/h0036316.

[brb371603-bib-0017] Giannousi, Z. , I. Manaras , V. Georgoulias , and G. Samonis . 2010. “Illness Perceptions in Greek Patients with Cancer: A Validation of the Revised‐Illness Perception Questionnaire.” Psycho‐Oncology 19, no. 1: 85–92. 10.1002/pon.1538. 19189280

[brb371603-bib-0018] Hagger, M. S. , and S. Orbell . 2003. “A Meta‐Analytic Review of the Common‐Sense Model of Illness Representations.” Psychology & Health 18, no. 2: 141–184. 10.1080/088704403100081321.

[brb371603-bib-0019] Hagger, M. S. , and S. Orbell . 2005. “A Confirmatory Factor Analysis of the Revised Illness Perception Questionnaire (IPQ‐R) in a Cervical Screening Context.” Psychology & Health 20, no. 2: 161–173. 10.1080/0887044042000334724.

[brb371603-bib-0020] Herzog, K. , F. Schepper , R. Kamm‐Thonwart , et al. 2024. “Trajectories of Illness Perceptions in Paediatric Cancer Patients and Their Parents and Associations with Health‐Related Quality of Life: Results of a Prospective‐Longitudinal Study.” Psycho‐Oncology 33, no. 3: e6332. 10.1002/pon.6332. 38520473

[brb371603-bib-0021] Huang, W. , L. Zhang , and J. Yan . 2019. “Psychometric Evaluation of the Chinese Version of the Revised Illness Perception Questionnaire for Breast Cancer‐Related Lymphedema.” European Journal of Cancer Care 28, no. 1: e12900. 10.1111/ecc.12900. 30144206

[brb371603-bib-0022] Hvidberg, L. , L. F. Jensen , A. F. Pedersen , A. R. Aro , and P. Vedsted . 2014. “Measurement Properties of the Danish Version of the Illness Perception Questionnaire–Revised for Patients with Colorectal Cancer Symptoms.” Journal of Health Psychology 19, no. 10: 1279–1290. 10.1177/1359105313488978. 23818508

[brb371603-bib-0023] Kakemam, E. , E. Navvabi , A. H. Albelbeisi , F. Saeedikia , A. Rouhi , and S. Majidi . 2022. “Psychometric Properties of the Persian Version of Depression Anxiety Stress Scale‐21 Items (DASS‐21) in a Sample of Health Professionals: a Cross‐Sectional Study.” BMC Health Services Research 22, no. 1: 111. 10.1186/s12913-022-07514-4. 35078477 PMC8789546

[brb371603-bib-0024] Langford, L. , G. Latchford , and M. Mulvey . 2024. “Can Illness Representations be Used to Understand Pain Experienced in Breast Cancer Survivorship—A Cross‐Sectional Study.” Journal of Cancer Survivorship 19: 1080–1089. 38285112 10.1007/s11764-024-01533-2

[brb371603-bib-0025] Leventhal, H. 1980. “The Common Sense Representation of Illness Danger.” Contributions to Medical Psychology 2: 7.

[brb371603-bib-0026] Leventhal, H. , D. R. Nerenz , and D. J. Steele . 2020. “Illness Representations and Coping with Health Threats.” In Handbook of Psychology and Health 219–252. Routledge.

[brb371603-bib-0027] Lovibond, S. H. 1995. Manual for the Depression Anxiety Stress Scales. Sydney Psychology Foundation.

[brb371603-bib-0028] Meder, J. , and J. Didkowska . 2011. Podstawy Onkologii Klinicznej. CMKP.

[brb371603-bib-0029] Moss‐Morris, R. , K. Humphrey , M. H. Johnson , and K. J. Petrie . 2007. “Patients' Perceptions of Their Pain Condition across a Multidisciplinary Pain Management Program.” Clinical Journal of Pain 23, no. 7: 558–564. 10.1097/AJP.0b013e318093fcab. 17710004

[brb371603-bib-0030] Moss‐Morris, R. , J. Weinman , K. Petrie , R. Horne , L. Cameron , and D. Buick . 2002. “The Revised Illness Perception Questionnaire (IPQ‐R).” Psychology & Health 17, no. 1: 1–16. 10.1080/08870440290001494.

[brb371603-bib-0031] Motamed, N. , A. R. Ayatollahi , N. Zare , and A. Sadeghi Hassanabadi . 2005. “Validity and Reliability of the Persian Translation of the SF‐36 Version 2 Questionnaire.” EMHJ‐Eastern Mediterranean Health Journal 11, no. 3: 349–357. 16602453

[brb371603-bib-0032] Ogińska‐Bulik, N. 2016. “Negatywne i Pozytywne Następstwa Doświadczonej Traumy–rola ruminacji.” Psychiatria i Psychologia Kliniczna 16, no. 3: 182–187.

[brb371603-bib-0033] Oudin Doglioni, D. , A. L. Pham‐Hung D'Alexandry D'Orengiani , F. Galactéros , and M. C. Gay . 2022. “Psychometric Characteristics of the Revised Illness Perception Questionnaire (IPQ‐R) in Adults With Sickle Cell Disease.” Health Psychology and Behavioral Medicine 10, no. 1: 60–80. 10.1080/21642850.2021.2016411. 34993006 PMC8725854

[brb371603-bib-0034] Pasternak, A. , M. Poraj‐Weder , and K. Schier . 2021. “Polish Adaptation and Validation of the Revised Illness Perception Questionnaire (IPQ‐R) in Cancer Patients.” Frontiers in Psychology 12: 612609. 10.3389/fpsyg.2021.612609. 34054639 PMC8155706

[brb371603-bib-0035] Perneger, T. V. , A. Leplège , J. F. Etter , and A. Rougemont . 1995. “Validation of a French‐Language Version of the MOS 36‐Item Short Form Health Survey (SF‐36) in Young Healthy Adults.” Journal of Clinical Epidemiology 48, no. 8: 1051–1060. 10.1016/0895-4356(94)00227-H. 7775992

[brb371603-bib-0036] Petrie, K. J. , L. D. Cameron , C. J. Ellis , D. Buick , and J. Weinman . 2002. “Changing Illness Perceptions after Myocardial Infarction: an Early Intervention Randomized Controlled Trial.” Biopsychosocial Science and Medicine 64, no. 4: 580–586. 10.1097/00006842-200207000-00007 12140347

[brb371603-bib-0037] Piotrowicz, M. , and D. Cianciara . 2011. “Teoria Salutogenezy–nowe Podejście Do Zdrowia i Choroby.” Przegląd Epidemiologiczny 65, no. 3: 521–527. 22184959

[brb371603-bib-0038] Polit, D. , and C. Beck . 2020. Essentials of Nursing Research: Appraising Evidence for Nursing Practice. Lippincott Williams & Wilkins.

[brb371603-bib-0039] Reichardt, J. , A. Ebrahimi , H. Nasiri Dehsorkhi , et al. 2018. “Why Is This Happening to Me?–A Comparison of Illness Representations between Iranian and German People with Mental Illness.” BMC Psychology 6, no. 1: 33. 10.1186/s40359-018-0250-3. 30029696 PMC6053818

[brb371603-bib-0040] Rivera, E. , K. Levoy , C. Park , et al. 2024. “Internal Consistency Reliability of the Revised Illness Perceptions Questionnaire: A Systematic Review and Reliability Generalization Meta‐Analysis.” Journal of Health Psychology 29, no. 7: 734–746. 10.1177/13591053231221351. 38314719 PMC13131025

[brb371603-bib-0041] Rourke, M. T. , W. L. Hobbie , L. Schwartz , and A. E. Kazak . 2007. “Posttrauamatic Stress Disorder (PTSD) in Young Adult Survivors of Childhood Cancer.” Pediatric Blood & Cancer 49, no. 2: 177–182. 10.1002/pbc.20942. 16862538

[brb371603-bib-0042] Santos, C. , J. Pais‐Ribeiro , and C. Lopes . 2003. “The “Revised Illness Perception Questionnaire” (IPQ‐R) Adaptation and Validation on Cancer Patients.” Arquivos de Medicinea 17, no. 4: 136–187.

[brb371603-bib-0043] Scharloo, M. , R. J. Baatenburg de Jong , T. P. Langeveld , E. van Velzen‐Verkaik , M. M. Doorn‐op den Akker , and A. A. Kaptein . 2005. “Quality of Life and Illness Perceptions in Patients with Recently Diagnosed Head and Neck Cancer.” Head & Neck 27, no. 10: 857–863. 10.1002/hed.20251. 16114002

[brb371603-bib-0044] Schoormans, D. , L. Wijnberg , H. Haak , O. Husson , and F. Mols . 2020. “Negative Illness Perceptions Are Related to Poorer Health‐Related Quality of Life among Thyroid Cancer Survivors: Results from the PROFILES Registry.” Head & Neck 42, no. 9: 2533–2541. 10.1002/hed.26290. 32488948 PMC7496500

[brb371603-bib-0045] Vegni, E. , D. Gilardi , S. Bonovas , et al. 2019. “Illness Perception in Inflammatory Bowel Disease Patients Is Different Between Patients with Active Disease or in Remission: a Prospective Cohort Study.” Journal of Crohn's and Colitis 13, no. 4: 417–423. 10.1093/ecco-jcc/jjy183. 30517669

[brb371603-bib-0046] Ware, J. E., Jr. , and C. D. Sherbourne . 1992. “The MOS 36‐Ltem Short‐Form Health Survey (SF‐36).” Medical Care 30, no. 6: 473–483. 10.1097/00005650-199206000-00002. 1593914

[brb371603-bib-0047] Watson, D. , L. A. Clark , and A. Tellegen . 1988. “Development and Validation of Brief Measures of Positive and Negative Affect: The PANAS Scales.” Journal of Personality and Social Psychology 54, no. 6: 1063–1070. 10.1037/0022-3514.54.6.1063. 3397865

[brb371603-bib-0048] Weinman, J. , K. J. Petrie , R. Moss‐Morris , and R. Horne . 1996. “The Illness Perception Questionnaire: A New Method for Assessing the Cognitive Representation of Illness.” Psychology & Health 11, no. 3: 431–445. 10.1080/08870449608400270.

